# Motion and Sash Height (MASH) alarms for efficient fume hood use

**DOI:** 10.1038/s41598-021-00772-y

**Published:** 2021-11-01

**Authors:** Johnathan Kongoletos, Ethan Munden, Jennifer Ballew, Daniel J. Preston

**Affiliations:** 1 Lab Energy Assessment Center, Massachusetts Institute of Technology, 32 Vassar St., Cambridge, MA 02139 USA; 2 Department of Architecture, Massachusetts Institute of Technology, 77 Massachusetts Ave., Cambridge, MA 02139 USA; 3 Department of Mechanical Engineering, Massachusetts Institute of Technology, 77 Massachusetts Ave., Cambridge, MA 02139 USA; 4 Department of Mechanical Engineering, Rice University, 6100 Main St., Houston, TX 77005 USA

**Keywords:** Energy and society, Psychology and behaviour, Sustainability

## Abstract

Ventilation, including fume hoods, consumes 40–70% of the total energy used by modern laboratories. Energy-conscious fume hood usage—for example, closing the sash when a hood is unused—can significantly reduce energy expenditures due to ventilation. Prior approaches to promote such behaviors among lab users have primarily relied on passive feedback methods. In this work, we developed a low-cost fume hood monitoring device with active feedback to alert lab users when a fume hood is left open and unused. Using data collected by the building management system, we observed a 75.6% decrease in the average sash height after installation of these “Motion and Sash Height” (MASH) alarms, which would result in a reduction roughly equal to 43% of the annual carbon emissions of a typical American vehicle, for each fume hood. The MASH alarm presented here reduced energy costs by approximately $1,159 per year, per hood, at MIT.

## Introduction

Fume hoods maintain the quality of air in laboratory workspaces while conducting experiments with the potential to release toxic fumes. Hoods also serve as a barrier between lab users and potential spills, splashes, fires, or explosions involving hazardous chemicals that may occur during an experiment. This physical protection is provided by the sash, a transparent window which often slides vertically at the front of a hood to allow access. Meanwhile, air quality in the greater lab space is maintained as hoods constantly pull air inwards (beneath and around the sash), and expel this air out of the building through an exhaust system. Removing the large quantities of air drawn through fume hoods within a laboratory subsequently places a burden on the building heating, ventilation, and air conditioning (HVAC) system, forcing the system to adjust to maintain the desired temperature, humidity, and other variables related to human comfort and providing a controlled environment for experiments. The energy required for these adjustments quickly adds up, consuming 40–70% of the total energy used in modern laboratories^1–3^. Where some attempts have been made to move towards horizontal sash hoods for energy savings, the research community prefers the ergonomics of a vertical sash and vertical sashes contain better than horizontal sashes at the same face velocity^4^. This then leaves them largely unchanged for the past 60 years^5,6^. Some literature proposes short-term shutdowns of the fume hoods when not in use^7^, but there are inherent risks to users and building occupants without proper decontamination and communication, both of which are medium to long-term approaches.

A better option is to use variable air volume (VAV) fume hoods, which account for 67% of hoods in the United States^8^, exhaust air at a flow rate proportional to the height of their sashes. There have been several attempts to reduce the amount of energy that is wasted by VAV fume hoods by reducing the average height of the sashes of these hoods. A program at the Massachusetts Institute of Technology (MIT) provided lab users with monthly feedback on their lab groups’ performance in keeping sashes closed in their lab spaces, and resulted in a 26% decrease in the average sash height^9^. At Harvard, “Shut the Sash” stickers were placed on fume hoods and used in conjunction with automatic sash closers to reduce the HVAC energy consumption by 70%^2,10^. A study at UC Davis tested the effects of similar stickers and found they saved $1,300 annually, per hood, in energy cost savings^11^. The passive methods used in these studies did not directly encourage user behavioral change at the time of the behavior to be changed, however; in contrast, feedback closer in time to the behavior or action to be changed has been reported to be more effective^9,12^. Further, a study from the University of Toronto found that passive methods had little long-term impacts on behaviors^13^.

More recent research at MIT showed that the use of active feedback, in the form of auditory alarms, significantly reduces the amount of energy wasted by fume hoods^8^. In that work, a device was developed to monitor the sash height of a fume hood and any motion occurring in front of the hood using a webcam and augmented reality (AR) tags attached to the hood. This device was programmed to notify users when they left the hood unattended and neglected to shut the sash. The device reduced wasted energy (defined as excess energy consumed as a result of the hood being open when not in use) by 87–98%. It cost $264 per device and saved labs $358 per hood, on average, in annual energy costs. One of the main drawbacks of this device, however, was its cost: despite having a payback period of less than one year, the “large” initial investment was hypothesized to potentially prevent labs or facilities departments from investing in this energy management solution. In addition, this device required an extensive setup procedure and had a relatively large footprint (approximately 10 × 10 × 30 cm). Finally, the AR tags mounted on the hood could interfere with lab work, or, alternatively, lab users could interfere with the AR tags, detrimentally impacting the performance of the device. Therefore, this work seeks to fill a research gap through the development and proof of concept of a smaller, less expensive device with a simplified setup procedure that produces the same feedback when a hood is open and not in use would overcome these issues while still offering the desired energy-saving benefits. This work then seeks to validate energy savings through real-world implementation and results.

## Methods

In this work, we developed an inexpensive (less than $20) fume hood monitoring device, called the Motion and Sash Height (MASH) alarm, that provides active feedback to lab users when fume hoods are left open and not in use. This device does not store data, and, in contrast to the use of live processing of a video stream of the fume hood to collect data used in prior work^8^, it requires only a passive infrared (PIR) motion sensor and a magnetic reed switch (a type of limit switch) to determine whether users are present and if the sash has been left open. We mounted these MASH alarms on 17 fume hoods (the “test” group), and we monitored these hoods, as well as nine hoods without MASH alarms installed but co-located in rooms that did have MASH alarms on other hoods (the “influenced” group), and 19 hoods in rooms without any alarms installed (the “control” group). All of these hoods were monitored by a pre-existing building management system, which recorded their instantaneous sash heights over time. We found that hoods in the test group exhibited a 76% average reduction in sash height compared to a reduction of only 10% in the control group. Surprisingly, we found that the hoods in the influenced group also exhibited a 71% reduction, suggesting that significant changes in user behavior can be attained even without use of MASH alarms on every fume hood within a given lab (possibly by raising lab user awareness). The average reduction in sash height for the test group of hoods corresponds to a predicted annual electrical energy savings of 3734 kWh (13,442 MJ), steam savings of 70.0 klbs (71,644 MJ), and chilled water savings of 1997 ton-hours (25,283 MJ) per hood with the MASH alarms installed, which is equivalent to a monetary savings of $1159 per year and a reduction of 2.0 metric tons of CO_2_ emissions per year. This analysis assumes constant energy-based utility pricing effective for MIT in 2018^14^.

**Figure 1:**
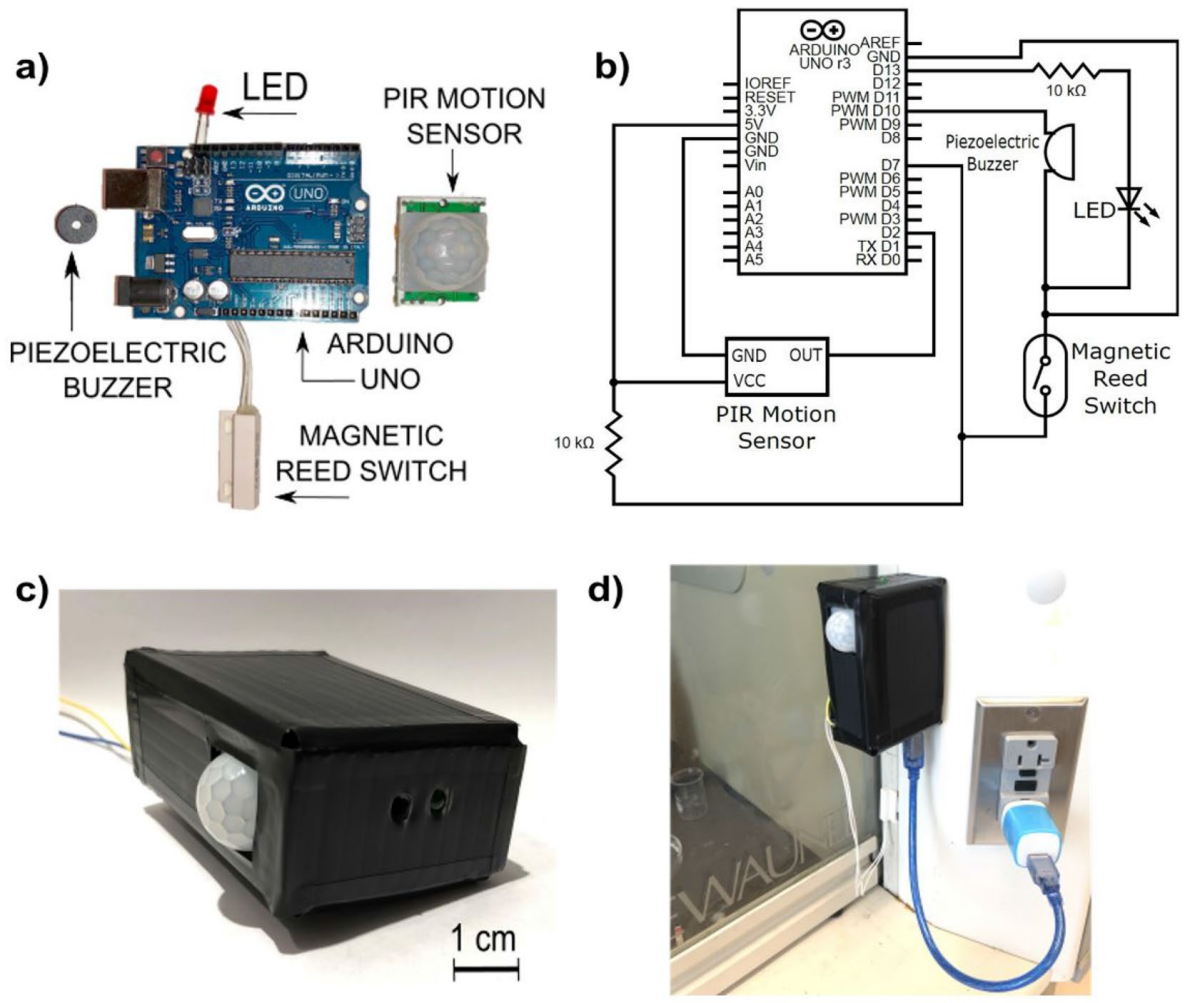
The fume hood monitoring device. The device is composed of an Arduino UNO R3 microcontroller board with off-the-shelf peripherals (**a**) connected as shown (**b**). The assembled device is encased in a corrugated plastic enclosure (**c**) and mounted to one side of the fume hood sash (**d**).

**Figure 2:**
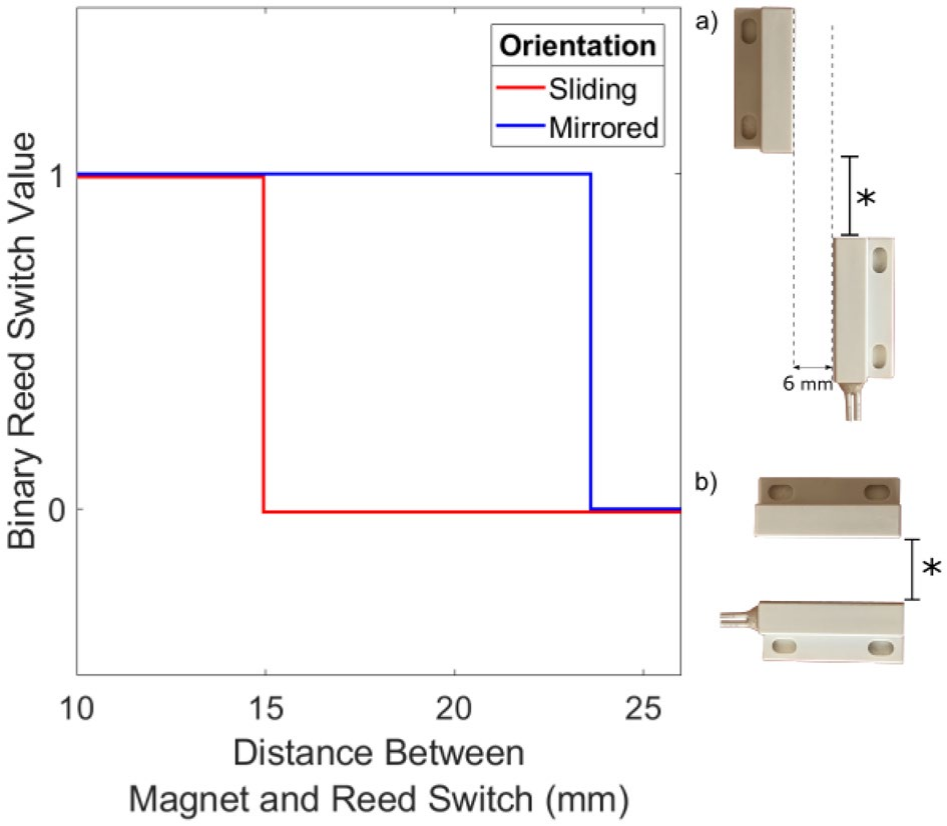
Magnetic reed switch characterization. Based on ten magnetic reed switches and maintaining a 6 mm clearance in the mirrored case, the hood sash is reported to be closed at a separation distance of 15.0 mm in the sliding case (**a**), and 23.6 mm in the mirrored orientation (**b**).

**Figure 3:**
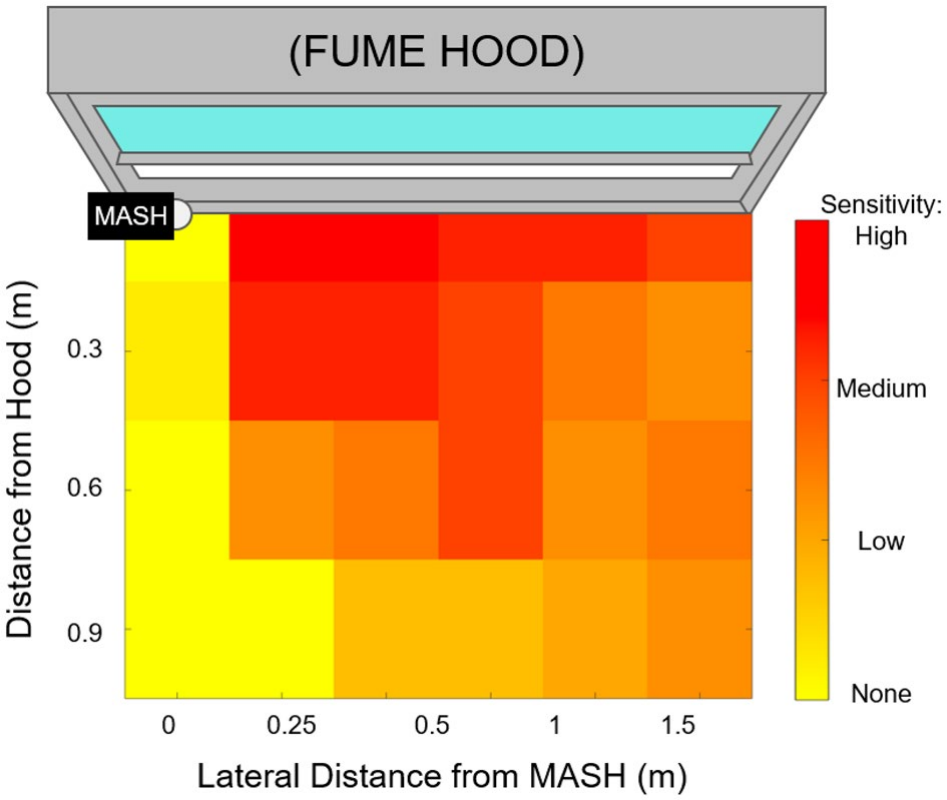
PIR motion sensor characterization. 1.2 m (four feet) above the ground and in front of the fume hood, PIR motion sensors produced repeatable results and are suitable for use in this application. Qualitatively, “high” sensitivity indicates that only a small movement by a human user can be sensed (a small motion of a finger), while “low” sensitivity indicates that a large movement (waving a hand) is required for the sensor to detect motion. The sensor should be positioned away from common walking routes to avoid false motion detections.

We constructed the MASH alarm using an Arduino UNO R3 microcontroller as the main processor. We attached a magnetic reed switch, a passive infrared (PIR) motion sensor, a piezoelectric buzzer, and an LED to the Arduino UNO (Fig. 1). We used the magnetic reed switch to determine if the sash of a hood was open. The reed switch was attached to the side of the frame of the hood, and a small magnet was attached to the movable sash of the hood and aligned next to the reed switch such that the circuit would close when the sash was fully shut, and the circuit would open when the sash was lifted (due to misalignment between the magnet and the reed switch). The reed switch behavior was characterized in both sliding (Fig. 2a) and mirrored (Fig. 2b) configurations as a function of sash height; we used the sliding configuration in this study. To detect if a lab user was present at the hood, we relied on the PIR motion sensor, which could detect any movement that occurred directly in front of the hood (we experimentally characterized the sensitivity of this component in detail, shown in Fig. 3). In addition to these modules, we added a piezoelectric buzzer and LED to indicate when the alarm was active, i.e., when a lab user had unnecessarily left the hood open for an extended period of time while not in use. The enclosure of the alarm was made from a sheet of corrugated plastic (fluted polypropylene) folded into a 5.5 × 8.4 × 3.2 cm box, and the enclosure contained the Arduino UNO, PIR motion sensor, buzzer, and LED. This enclosure was designed with openings to (1) supply power to the Arduino UNO, (2) update the program on the Arduino UNO, (3) allow the motion sensor to monitor the surroundings, and (4) feed the wires to the magnetic reed switch.

The MASH alarm continuously checks whether the magnetic reed switch is open and if there is motion detected by the PIR motion sensor. This program loops indefinitely as long as the device is powered. If the magnetic reed switch has been left open and there has been no motion detected by the motion sensor for a specified amount of time, the alarm will sound and the LED will flash. The amount of time until the alarm sounds is an adjustable variable in the program (as described in the Supplementary Information, Section S1). We polled a portion of the labs that would be involved in the experiment, and we found that a three-minute delay would be satisfactory (i.e., unobtrusive) for most of the labs. Further work can be done to assess the variation in energy savings as a result of changing the delay, or the influence of a variable delay dependent on the type of lab research being conducted, may be beneficial, and could represent a useful collaboration with behavioral scientists; such work was not included within the scope of this research.

We installed MASH alarms on fume hoods in Building 56 at MIT. The research conducted in Building 56 falls predominantly under the biology, chemistry, and chemical engineering departments, and there are a total of 45 variable air volume hoods operating in the building. We installed MASH alarms on 17 fume hoods (the “test” group). A second group consisted of 19 hoods that did not receive MASH alarms, were not affiliated with the labs or research groups involved in our test, and were not in the same rooms as any of the MASH alarms (the “control” group). Finally, nine hoods were either in the same room as a MASH alarm, or were used by a lab or research group that received some number of MASH alarms in other rooms in their lab space (the “influenced” group). All MASH alarms were installed on September 17, 2018, with the exception of one MASH alarm that was installed on September 14.

**Figure 4:**
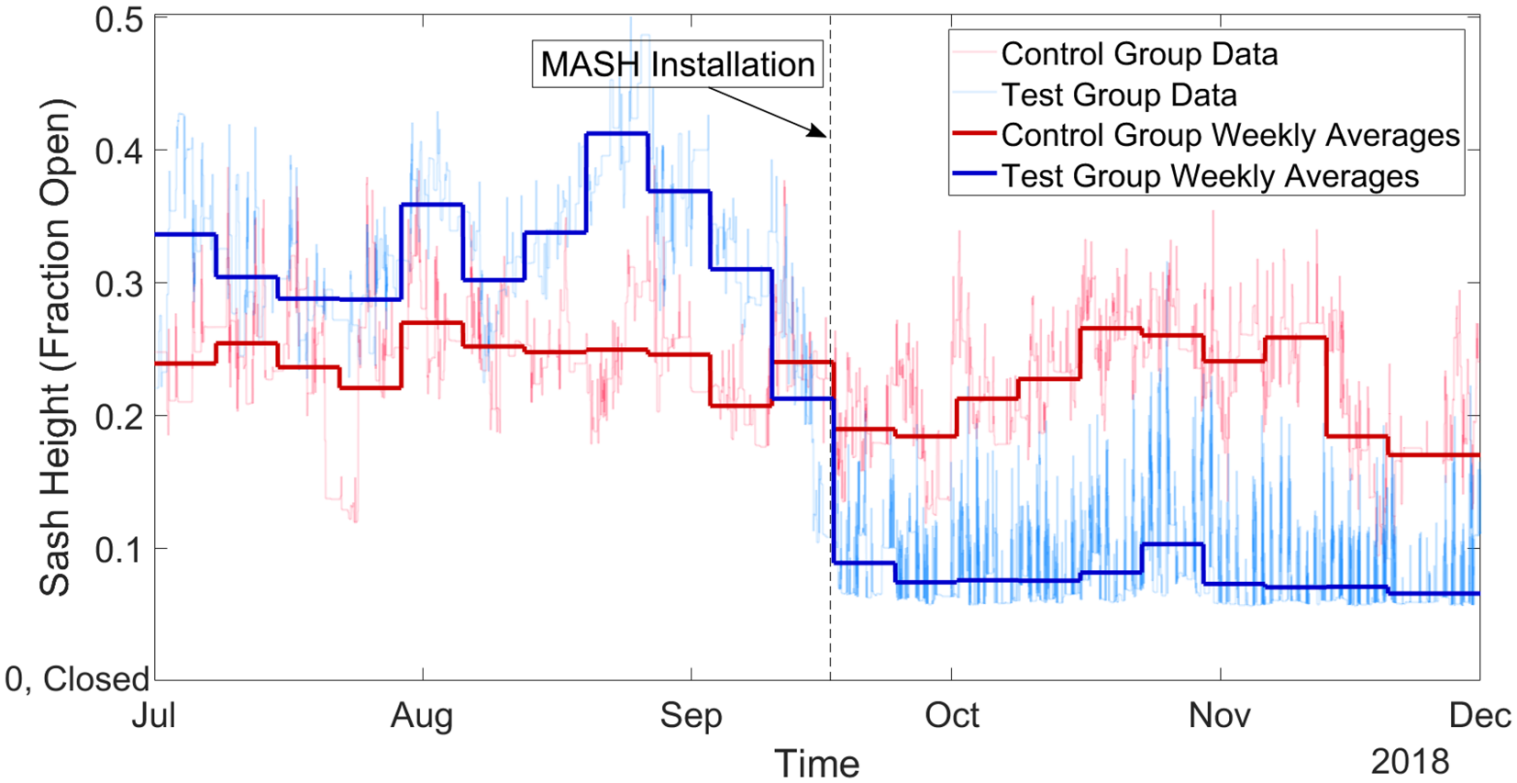
Test hoods versus control hoods. This figure shows the average sash heights of the test and control groups, as recorded every 15 min (lighter colored lines), overlaid with the weekly average sash heights of both groups (bold). After the installation of the MASH alarms, the sash heights of the control group remained relatively unchanged, but the test group exhibited a significant drop in average sash heights. (A value of 0.07 or below corresponds to a closed fume hood sash in the KGS Clockworks system).

**Figure 5:**
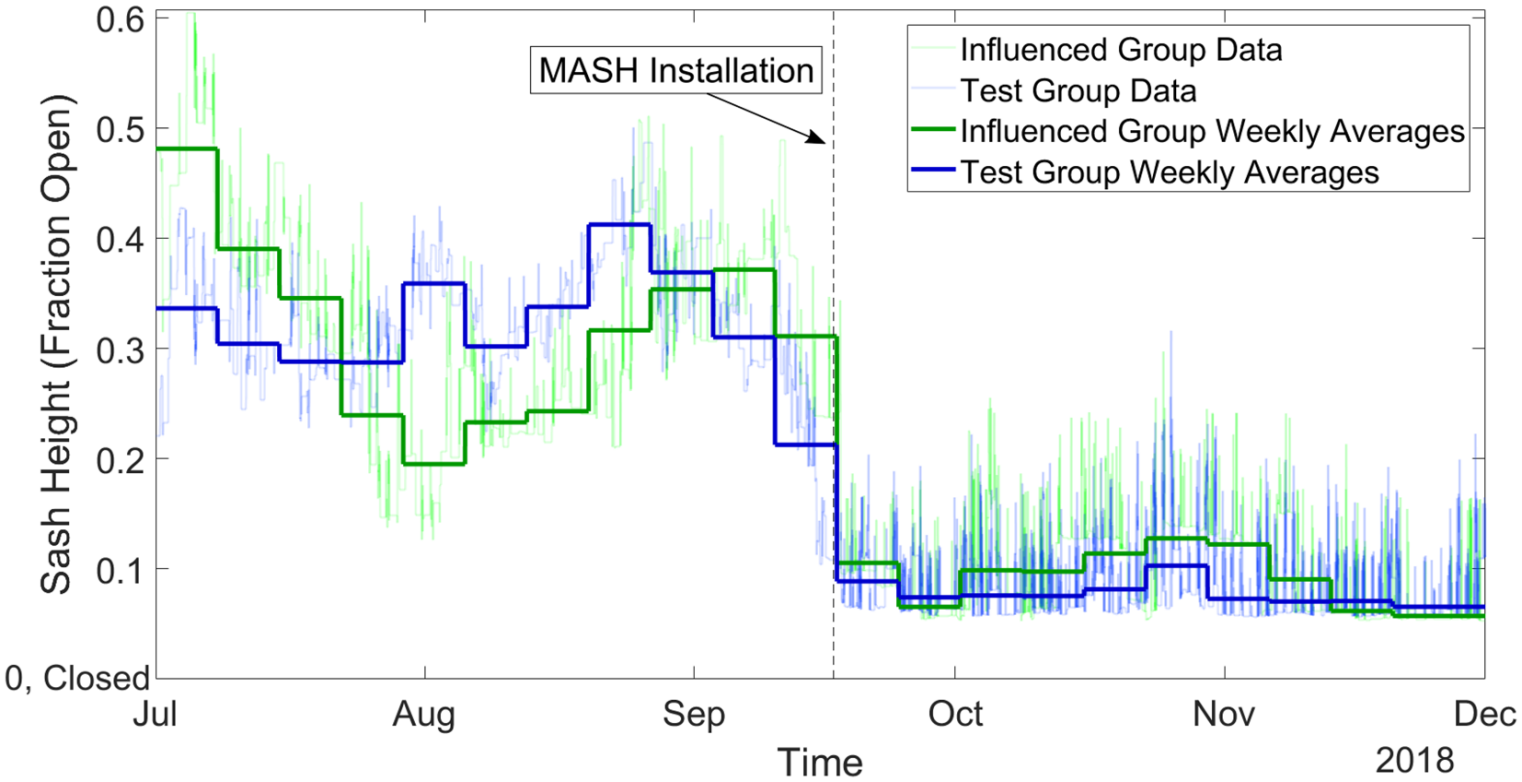
Test hoods versus influenced hoods. This figure shows the average sash heights of the test and influenced groups recorded every 15 min (lighter colored lines) and the weekly average sash heights of both groups (bold). The dashed line indicates when the MASH alarms were installed. Despite not having an alarm attached to the fume hood itself, having an alarm co-located in the lab caused the average sash height of the “influenced” hoods to decrease in a similar manner to the test group. This evidence shows that having an alarm near a hood, or having knowledge that the alarms are installed on other hoods in the lab, can cause lab users to operate their hoods in a more energy efficient manner. (A value of 0.07 or below is considered closed by the KGS clockworks system).

To collect sash height data, we retrieved data from KGS Buildings’ Clockworks system. Clockworks is a data aggregator and archival system employed by MIT to collect and store data from individual buildings’ building management systems, including chilled water usage, steam usage, and fan power, among other data. We used data collected by the sash height sensors associated with the fume hoods that we considered in this study. Through Clockworks, this data was available in five-minute point data; we used this data to compare the average sash heights during the span of the 11 weeks before the alarms were installed (July 1 through September 16, 2018), with the 11 weeks during which the alarms were active (September 16 through December 1, 2018) (Figs. 4, 5). We also averaged the five-minute point data to weekly average sash height values to aid visualization of the data; a reduced weekly sash height indicates that the hoods were left open for less time throughout the week, implying that lab users were shutting the sash more consistently when they left their workspace unattended, and thereby saving energy^15^.

## Results and discussion

From the sash height, we can estimate the amount of energy consumed by the fume hood based on the area of the sash opening and the rate at which the hood pulls air, i.e., the face velocity. A calibrated TSI VelociCalc 9535 Air Velocity Meter was used by a MIT health and safety representative to measure the face velocity at each fume hood and the results reported to the team. Fume hoods require roughly 1.8 watts per CFM (cubic feet per minute) of airflow that is pulled through the hood and exhausted^16^. In our experiment, we averaged the values of the sash width, sash height, and face velocity for each group to determine representative hood characteristics for each of the three groups. We found the representative sash width and face velocity by averaging the measured values of the hoods in each of the three groups, which ultimately provides the quantitative average energy consumption on a per-hood bases for each group of hoods by the commutative property of multiplication. To calculate values for the average sash height before and after installation of the MASH alarm in each group, we used the Clockworks data. By multiplying the average sash height by the average sash width and face velocity, we calculated the average CFM of the hoods in each of the three groups (Eq. 1). We found the control group to have an average of 250 CFM (0.118 m^3^/s) before the installation of the MASH alarms, which decreased slightly to an average of 227 CFM (0.107 m^3^/s) after the alarms were installed elsewhere in Building 56 (Fig. 4). The test group was found to have an average of 313 CFM (0.148 m^3^/s) before the MASH alarms were installed, which decreased by over 75% to an average of 77 CFM (0.04 m^3^/s) after the alarms were installed (Fig. 4). The influenced group exhibited an average of 362 CFM (0.171 m^3^/s) before the MASH alarms were installed and an average of 105 CFM (0.05 m^3^/s) after the alarms were installed (Fig. 5). We used this data to calculate the average energy consumption per hood for each group in our study by multiplying the average airflow rate (in CFM) by 1.8 W/CFM (Eq. 2). This value represents the average power draw from a hood in each group over the period we collected the data, in watts. We used electrical energy units of kWh/yr for easier comparison with other literature. A full year (8760 h) is used for the duration of time since the fume hoods run continuously unless the hoods, and their associated ductwork, have been decontaminated and the room rebalanced to ensure required air change rates are maintained, which happens rarely and typically only when a hood is being permanently decommissioned.

1 $$ Volumetric\;Air\;Flow \left[ {CFM} \right] = \left( {sash\;height \left[ {{\text{ft}}} \right]} \right) x \left( {sash\;width \left[ {{\text{ft}}} \right]} \right) x \left( {face\;velocity \left[ {{\text{ft}}/{\text{min}}} \right]} \right) $$

2 $$ Electric\;Energy\;for \;Fans \left[ {\text{kWh/year}} \right] = CFM \times 1.8\frac{W}{CFM} \times \frac{{1 \;{\text{kW}}}}{{1000 \;{\text{W}}}} \times \frac{{8760\;{\text{h}}}}{{1\;{\text{year}}}} $$

Similarly, for the heating and cooling loads, we used the outdoor air temperature and relative humidity from the TMY3 (Typical Meteorological Year, third collection) dataset for Boston Logan airport and assumed that the air was delivered to the space at a strict setpoint of 65 °F (18.3 °C) using a dewpoint-based control strategy with a 55 °F (12.8 °C) dewpoint and humidification to 50% relative humidity (RH). To accomplish this, the various enthalpy differences between stages in the air handler were calculated and added together as applicable. This process encompasses the sensible addition of half of the total fan power (assuming the supply and exhaust fan power are equally split), the initial preheating stage to preheat air to 35 °F (1.7 °C), the chilled water coil to cool and dehumidify the airstream to 55 °F (12.8 °C), the reheat stage to heat the air to 65 °F (18.3 °C), and the humidification stage to humidify air to a 50% RH minimum at 65 °F (18.3 °C). In the process, heating, cooling, and humidifcation are applied only as needed. For example, 40 °F (4.4 °C) outside air only experiences heating impact when passing over the reheat coil. Likewise, 55 °F (12.8 °C) and 100% RH air does not receive or give up heat to any heat exchangers while passing through the air handler. At 65 °F (18.3 °C), the 55 °F (12.8 °C) and 100% RH air would be equivalent to 70% RH. On a unitary basis, each CFM (0.028 m^3^/min) annually consumes 296 lbs (302.4 MJ equivalent) of steam and 8.43 ton-hours (106.7 MJ equivalent) of chilled water for the Boston climate under this control strategy.

**Figure 6:**
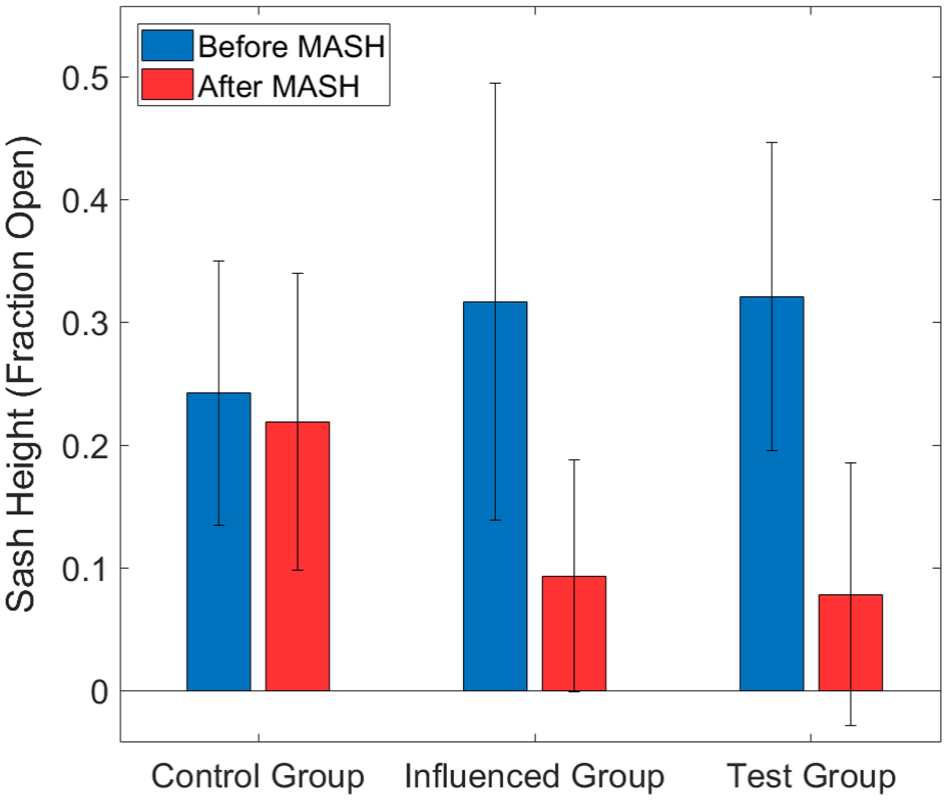
Summary of results. Average sash heights are shown before and after MASH installation, with error margins based off of a 95% confidence interval. The average sash height decreased among all groups. With the 95% confidence interval, the test group’s average sash height decreased with statistical significance (described in the Supplementary Information, Section S4).

Our experiment demonstrates that hoods with a MASH alarm installed exhibit a decrease in average sash heights of over 75%. Additionally, the hoods in the influenced group, co-located in labs with MASH alarms present but without MASH alarms installed, experienced a decrease in average sash height of over 70%. Meanwhile, the average sash height of the control group did not change significantly; in the control group, the average sash height decreased by only 10%. These results are summarized in Fig. 6. It should be noted that the baseline case was a situation in which the fume hoods already had the customary “Shut the Sash” stickers, but did not have a laboratory manager actively monitoring the sash heights in either the pre or post-installation cases. Further laboratory management involvement may be helpful.

We calculated a 95% percent confidence interval on the mean sash heights before and after installing the MASH alarm across the hoods in each group to determine if the average sash heights changed significantly due to the presence of the alarm (details included in the Supplementary Information, Section S4). We found that, at a confidence level of 95%, the decrease in the average sash height in the control group was not statistically significant, while the difference in the test group was significant (Fig. 6). The influenced group’s decrease in sash height was more significant than that of the control group, but less than that of the test group. These results demonstrate that the presence of a combined auditory and visual alarm, used to provide real-time feedback, reminded lab users to shut the hood when not in use, leading the lab to use considerably less energy than it would have without the MASH alarm.

Fume hoods that were located in the same room as a MASH alarm had a similar, slightly smaller decrease in average sash height. This is likely due to the lab users either hearing the alarm or otherwise knowing that the experiment was occurring, which caused them to consciously (or subconsciously) try to close their sash when not in use. This confounding variable is a positive consequence of having at least one alarm installed in a laboratory. This effect should motivate research groups to install at least one MASH alarm in their laboratory.

The electrical energy saved in the experimental group (comparing the averages before and after installation of the MASH alarm and extrapolating to a per-annum basis) amounts to 3734 kWh/year per hood. Using data from the U.S. Environmental Protection Agency, this is equivalent to 2.6 metric tons of CO_2_ in carbon emissions, or around one third of what the average household produces in the same amount of time^17,18^. Using data specific to the MIT campus, this reduction would be equivalent to 2.0 metric tons CO_2_^14^_._ Having around one thousand VAV fume hoods in use, MIT would annually save 3734 MWh (13.44 × 10^6^ MJ) of electricity, 70,003 klbs (71.64 × 10^6^ MJ equivalent) of steam, and 2.0 million ton-hours (25.28 × 10^6^ MJ equivalent) of chilled water if MASH alarms were installed on every hood, equivalent to the annual energy consumption of around 1149 households^19^. Further, the CO_2_ savings from those one thousand VAVs would be equivalent to removing roughly 435 vehicles from the road^17^. As universities and research institutions across the country often have many fume hoods on site, the installation of MASH alarms could lead to significant energy savings for the country as a whole. While the sash height sensors vary from hood to hood, if one were to use the propagation of uncertainty approach based solely on the anemometer used^20^ and a commercially available sash height sensor^21^, the resultant measurement uncertainty would be on the order of ± 3%.

The financial incentives related to the installation of these devices are also convincing. Each device is fabricated using an Arduino UNO, simple commercially-available peripherals, and corrugated plastic, which results in a total cost of only $17.07 per device, and is more palatable than other potentially-risky strategies^22^. Since the device is inexpensive, the initial investment is miniscule compared to the yearly savings. At a calculated price of $4.89 per cfm-year, in close agreement with that presented by Mills and Sartor^6^ and 72% of that calculated by Wesolowski^9^, the projected savings per year, per hood, due to MASH alarm installation is $1159 (assuming electricity costs of $0.11 per kWh of electricity, $0.082/ton-hour of chilled water, and $8.346/klb of steam)^14^. Based on material costs, a MASH alarm has a payback period of 5.4 days. Further, the projected savings are likely to be conservative, given that the trigeneration system used at the MIT campus lowers the marginal price for each unit of energy. Savings will vary regionally depending on variations in utility pricing (costs) and weather (heat loads).

MASH alarms could be improved by making them smaller and even more cost effective. Smaller processors with similar capabilities to the Arduino UNO were explored, but ultimately the Arduino UNO was chosen due to convenience, and cost-effectiveness. A future iteration of the MASH alarm could use a smaller, cheaper processor in addition to removing the built-in breadboard and directly soldering the components together, which would increase the sturdiness of the device and lower the physical profile. The device could also use an ultrasonic distance sensor and a Wi-Fi module to track the sash height of the hood and log the data in addition to the alarm feature. This could be used to give labs real-time feedback on their overall performance, which has been shown to reduce the energy wasted by fume hood use^9^. This additional feature would add an upfront material cost of only $15, as these common Arduino parts are readily available for purchase.

## Conclusions

The MASH alarm is an easy-to-build, cost-effective energy management solution that could save labs across the country on the order of $1159 per hood, per year. This savings is given for utility pricing at MIT, and the cost savings estimate is a conservative value given that the MIT trigeneration system lowers the marginal cost of fuel. After the installation of the MASH alarms, a 75.6% decrease in the average sash height was observed, which resulted in a predicted annual savings of 3734 kWh (13,442 MJ) of electrical energy, 70.0 klbs (71,644 MJ) of steam, and 1997 ton-hours (25,283 MJ) of chilled water for each hood, as well as a reduction in carbon emissions by 2.0 metric tons CO_2_/year for each hood. The inexpensive (less than $20), open-source MASH alarm presented here is attractive due to both its potential financial benefits and its ability to reduce emissions and energy consumption in the face of global warming and depletion of nonrenewable resources.

In future work, this alarm could also be used to ensure that biosafety cabinets and differentially pressured rooms remain closed. Although biosafety these cabinets are most commonly CAV systems, ensuring that they are closed when not actively in use is of interest to health and safety personnel. For a room maintained at negative pressure, these alarms, when placed on the doorway, will help to ensure that a majority of the air provided to the space is treated and filtered. Further, MASH alarms can be easily adapted for horizontally-sliding sash VAV fume hoods through placing multiple reed switches in series for the various panes of the sash system, or to freezers and lab ovens, where there are energy, safety, and sample quality benefits. Planned future work will also include the installation of 59 MASH alarms on a newer building on MIT’s campus, with building-level energy savings results forthcoming within the next year; these results will help illustrate how the MASH alarm can enable the BMS to achieve ventilation turn down settings that are currently unattainable when hoods are left open.

## Supplementary Information

Supplementary Information.
