# Supplementary material for: Motion and Sash Height (MASH) alarms for efficient fume hood use
Source: Sci Rep. 2021 Nov 1;11:21412. doi: 10.1038/s41598-021-00772-y (PMC8560830; doi:10.1038/s41598-021-00772-y)
Supplement: Supplementary file 1 — Supplementary Information. [file 41598_2021_772_MOESM1_ESM.docx]

Supplementary Information for: **Motion and Sash Height (MASH) Alarms for Efficient Fume Hood Use**

Johnathan Kongoletos^1,2,†,^*, Ethan Munden^1,†^, Jennifer Ballew^1,^*, Daniel J. Preston^1,3,4^

^1^Lab Energy Assessment Center, Massachusetts Institute of Technology, 32 Vassar St., Cambridge, MA 02139, USA

^2^Dept. of Architecture, Massachusetts Institute of Technology, 77 Massachusetts Ave., Cambridge, MA 02139, USA

^3^Dept. of Mechanical Engineering, Massachusetts Institute of Technology, 77 Massachusetts Ave., Cambridge, MA 02139, USA

^4^Dept. of Mechanical Engineering, Rice University, 6100 Main St., Houston, TX 77005, USA

^†­­^ *Indicates equal contribution*

* *To whom correspondence may be addressed:*

John Kongoletos

*Email:* [jkongole@mit.edu](mailto:dpreston@mit.edu)

**Supplementary Information Table of Contents**

**S1.** Computer Code

**S2.** Financial Information

**S3.** Calculation of Confidence Intervals

**S4.** Calculation of Airflow

**S1. Computer Code.** For reproducibility, the code for the Arduino UNO installed on each MASH alarm is provided here, with comments provided after the “//” on each line.

const int alarm = 10; // alarm output on pin 10

const int limit = 7; // limit switch input on pin 7

const int motion = 2; // motion detector input on pin 2

const double alarmTime = 3; // time before alarm goes off if no motion detected and fume hood is open

int currentTime = 0; // dummy variable for moment when no motion detected

int elapseTime = 0; // dummy variable for elapsed time after motion not detected

int closed = 0;

int checkTime = 0;

int halfMinutes = 0; // workaround for the clock limitations of the Arduino UNO

double minutes = 0.0; // keeps track of minutes passed when the hood is left open

bool soundTheAlarm = 0;

bool alreadyAlarming = 0;

void setup() {

Serial.begin(9600);

pinMode(13, OUTPUT); // LED used for debugging purposes

pinMode(alarm, OUTPUT);

pinMode(limit, INPUT);

pinMode(motion, INPUT);

}

// function called to make the piezobuzzer alarm when the hood is left open

void alarmTone(int buzzer_pin){

digitalWrite(13, HIGH);

tone(buzzer_pin, 4500);

delay(100);

tone(buzzer_pin, 2500);

delay(100);

digitalWrite(13, LOW);

noTone(buzzer_pin);

delay(500);

}

// function called to make the buzzer sound when the sash is closed

void closeTone(int buzzer_pin){

tone(buzzer_pin, 3500);

delay(80);

tone(buzzer_pin, 5500);

delay(100);

noTone(buzzer_pin);

}

void loop() {

digitalWrite(13, LOW); // as long as LED is off, fume hood either closed or motion is detected

currentTime = millis(); // retrieves current time

alreadyAlarming = 0; // initialize the alarm in the "OFF" state

minutes = 0;

halfMinutes = 0;

if (!closed && digitalRead(limit) == LOW) // causes the buzzer to beep when the sash is closed

{

closeTone(alarm);

closed = 1;

}

else if (closed && digitalRead(limit) == HIGH) // change the value of "closed" when the sash is open

{

closed = 0;

}

while (digitalRead(limit) == HIGH and digitalRead(motion) == LOW) // while fume hood is OPEN and no motion detected

{

elapseTime = millis();

checkTime = elapseTime - currentTime; // amount of seconds that have passed since sash was left open and unattended

if (checkTime > 30000){ // counts every 30 seconds as workaround for clock limitations on the Arduino UNO

halfMinutes += 1;

currentTime = elapseTime; // reset the compared time

checkTime = 0;

}

minutes = halfMinutes/2.0 + checkTime/60000.0; // amount of minutes passed since sash left open and unattended

soundTheAlarm = alreadyAlarming or (minutes) > alarmTime;

if (soundTheAlarm) // if motion is not detected after alarmTime minutes

{

alreadyAlarming = 1;

alarmTone(alarm);

}

}

}

**S2. Financial Information.** Table S1 details the cost breakdown, by part, of a single MASH sensor; Table S2 describes calculation of the payback period of a MASH sensor.

**Table S1.** Cost breakdown of MASH alarm, noting the cost of each piece and the total cost to construct a single alarm.

| Part | Unit Cost ($) | Cost per Device ($) |
| --- | --- | --- |
| Arduino UNO R3 | 8.99 | 8.99 |
| Breadboard | 0.83 | 0.83 |
| Power Cube | 1.99 | 1.99 |
| PIR Sensor | 1.80 | 1.80 |
| Wires | 0.07 | 0.98 |
| Resistors | 0.15 | 0.45 |
| Piezo Buzzer | 0.56 | 0.56 |
| Magnetic Switch | 1.44 | 1.44 |
| LED | 0.03 | 0.03 |
| Total |  | **17.07** |

**Table S2.** Cost-benefit analysis of MASH alarm, including the amount of time the device pays itself back in energy cost savings (the simple payback period).

| Projected Annual Savings ($/yr) | 1,159 |
| --- | --- |
| Cost of MASH alarm ($) | 17.07 |
| Projected Payback Period (yrs) | 0.01 |
| Projected Payback Period (days) | 5.38 |

**S3. Calculation of Confidence Intervals.**

To determine the likelihood that the average sash heights decreased due to the presence of the MASH alarm, we used a 95% confidence interval on each sample to show how confident we are in the calculated means for each sample before and after the installation of the MASH alarm. Since our sample size for all three groups was less than 30, we used the Student’s t-distribution.

At a confidence level of 95%, the test values for each group is as follows:

*Test Group (16 degrees of freedom): t=2.120*

*Influenced Group (8 degrees of freedom): t=2.306*

*Control Group (18 degrees of freedom): t=2.101*

This led to our confidence intervals being constructed by:

$$True Average Sash Height=Calculated Average \pm t* \frac{s}{\sqrt{n}}$$

Where *t* is the test values based on the sample size of each group, *s* is the standard deviation of the average sash heights of the sample of hoods either before or after the MASH installation, and *n* is the number of hoods in each respective sample (Table S3).

**Table S3.** Confidence interval data for the three fume hood groups.

| Group | Calculated Average | Standard Deviation | Number of Hoods | Confidence Interval |
| --- | --- | --- | --- | --- |
| Test (Before MASH) | 0.318 | 0.244 | 17 | (0.192, 0.444) |
| Test (After MASH) | 0.078 | 0.208 | 17 | (-0.029, 0.185) |
| Influenced (Before) | 0.315 | 0.232 | 9 | (0.137, 0.493) |
| Influenced (After) | 0.091 | 0.123 | 9 | (-0.004, 0.186) |
| Control (Before) | 0.242 | 0.222 | 19 | (0.135, 0.349) |
| Control (After) | 0.219 | 0.251 | 19 | (0.098, 0.340) |

**S4. Calculation of Airflow.** The average airflow (CFM) for each group of fume hoods as shown in Table S4.

**Table S4.** Calculating CFM of hoods in each group (using Equation 1 from the main text).

|  | Test Group | Influenced Group | Control Group |
| --- | --- | --- | --- |
| Average sash width [in] | 50.59 | 50.80 | 48.00 |
| Average face velocity [ft/min] | 112.18 | 114.40 | 113.16 |
| Average sash height (before MASH) [in] | 7.95 | 8.97 | 6.62 |
| Average sash height (after MASH) [in] | 1.94 | 2.60 | 6.00 |
| Representative CFM (before MASH) [ft^3^/min] | 313.35 | 362.02 | 249.65 |
| Representative CFM (after MASH) [ft^3^/min] | 76.51 | 104.94 | 226.50 |
| Percent reduction in air volume [%] | 75.6 | 71.0 | 9.3 |
